# Supplementary material for: Altered microRNA Transcriptome in Cultured Human Liver Cells upon Infection with Ebola Virus
Source: Int J Mol Sci. 2021 Apr 6;22(7):3792. doi: 10.3390/ijms22073792 (PMC8038836; doi:10.3390/ijms22073792)
Supplement: Supplementary file 1 [file ijms-22-03792-s001.zip › Supplementary_File/C_ GO_Analysis_Results/16-30nt_go_Mayinga-96h-Huh7_vs_Control-96h-Huh7_up.mature_mirna_targets/CC_Pvalue_tree.pdf]

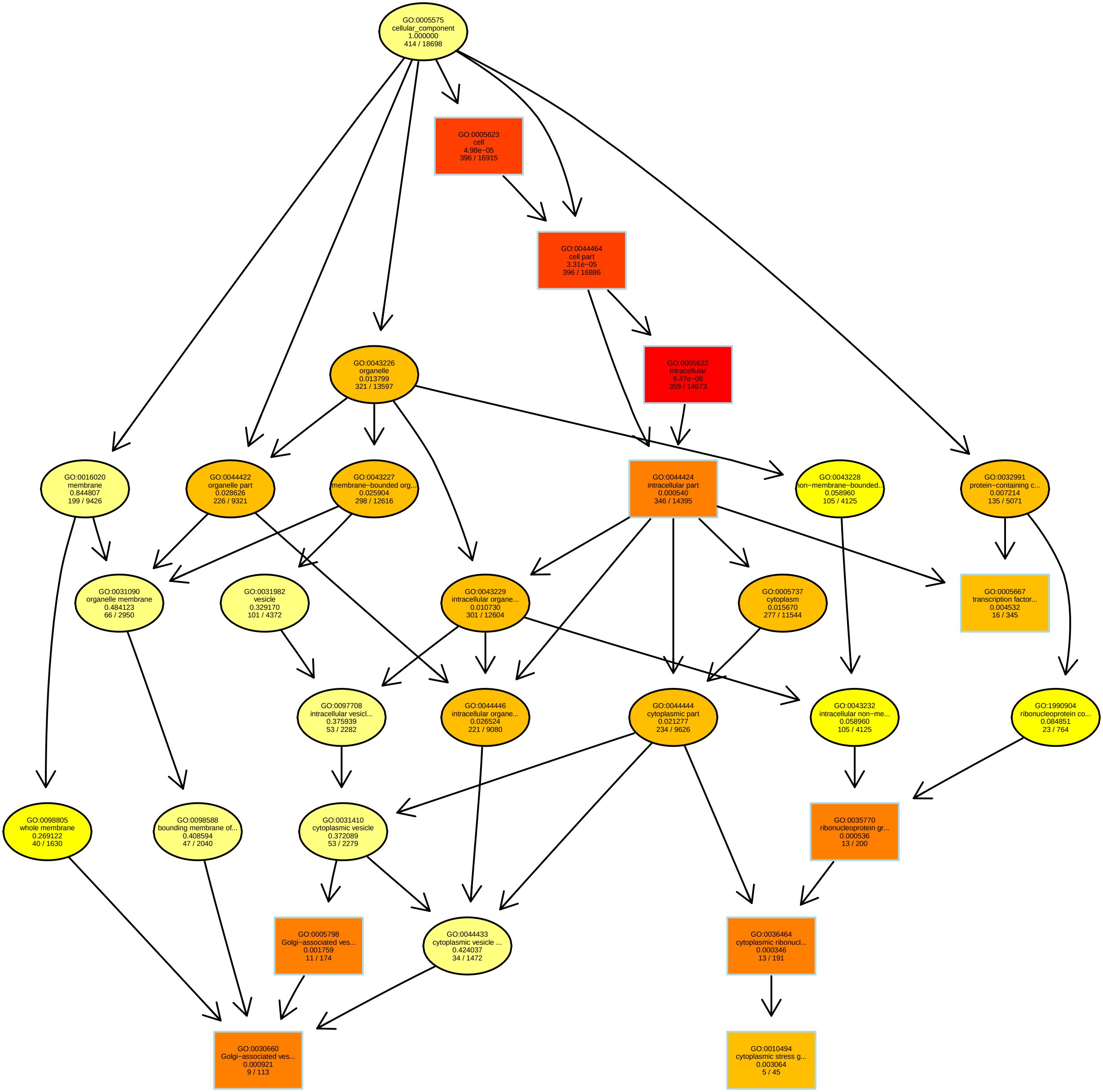
GO:0005575
cellular_component
1.000000
414 / 18698
GO:0005623
cell
4.98e−05
396 / 16915
GO:0044464
cell part
3.31e−05
396 / 16886
GO:0043226
organelle
0.013799
321 / 13597
GO:0005622
intracellular
9.47e−06
359 / 14673
GO:0016020
membrane
0.844807
199 / 9426
GO:0044422
organelle part
0.028626
226 / 9321
GO:0043227
membrane−bounded org...
0.025904
298 / 12616
GO:0044424
intracellular part
0.000540
346 / 14395
GO:0043228
non−membrane−bounded...
0.058960
105 / 4125
GO:0032991
protein−containing c...
0.007214
135 / 5071
GO:0031090
organelle membrane
0.484123
66 / 2950
GO:0031982
vesicle
0.329170
101 / 4372
GO:0043229
intracellular organe...
0.010730
301 / 12604
GO:0005737
cytoplasm
0.015670
277 / 11544
GO:0005667
transcription factor...
0.004532
16 / 345
GO:0097708
intracellular vesicl...
0.375939
53 / 2282
GO:0044446
intracellular organe...
0.026524
221 / 9080
GO:0044444
cytoplasmic part
0.021277
234 / 9626
GO:0043232
intracellular non−me...
0.058960
105 / 4125
GO:1990904
ribonucleoprotein co...
0.084851
23 / 764
GO:0098805
whole membrane
0.269122
40 / 1630
GO:0098588
bounding membrane of...
0.408594
47 / 2040
GO:0031410
cytoplasmic vesicle
0.372089
53 / 2279
GO:0035770
ribonucleoprotein gr...
0.000536
13 / 200
GO:0005798
Golgi−associated ves...
0.001759
11 / 174
GO:0044433
cytoplasmic vesicle ...
0.424037
34 / 1472
GO:0036464
cytoplasmic ribonucl...
0.000346
13 / 191
GO:0030660
Golgi−associated ves...
0.000921
9 / 113
GO:0010494
cytoplasmic stress g...
0.003064
5 / 45
